# Predicting the length of hospital stay of post-acute care patients in Taiwan using the Chinese version of the continuity assessment record and evaluation item set

**DOI:** 10.1371/journal.pone.0183612

**Published:** 2017-08-23

**Authors:** Chen-Yu Hung, Wei-Ting Wu, Ke-Vin Chang, Tyng-Guey Wang, Der-Sheng Han

**Affiliations:** 1 Department of Physical Medicine and Rehabilitation, National Taiwan University Hospital BeiHu Branch, Taipei, Taiwan; 2 Community and Geriatric Medicine Research Center, National Taiwan University Hospital BeiHu Branch, Taipei, Taiwan; 3 Department of Physical Medicine and Rehabilitation, National Taiwan University Hospital, Taipei, Taiwan; 4 Department of Physical Medicine and Rehabilitation, College of Medicine, National Taiwan University, Taipei, Taiwan; National Yang-Ming University, TAIWAN

## Abstract

**Background:**

The Chinese version of the Continuity Assessment Record and Evaluation (CARE-C) item set was developed to facilitate the assessment of post-acute care (PAC) patients in Taiwan. Considering that the length of hospital stay (LOS) has a significant effect on the total healthcare cost, determining whether the CARE-C scores could predict the LOS of PAC patients is of great interest to the PAC providers.

**Methods:**

This prospective trial included PAC patients with stroke or central nervous system injuries. The demographic data and CARE-C scores were collected after admission and before discharge. A multivariable stepwise linear regression model was used to identify the predictors of the LOS using age, sex, tube placement status, CARE-C component scores at admission, and score differences between admission and discharge as independent variables.

**Results:**

This study included 178 patients (66 women and 112 men), with a mean age of 61.9 ± 15.6 years. Indwelling urinary catheter placement status at admission (β = 0.241, p = 0.002) was a positive predictor of the LOS, whereas age (β = −0.189, p = 0.010), core transfer subscale score at admission (β = −0.176, p = 0.020), and difference in continence subscale score (β = −0.203, p = 0.008) were negative predictors of the LOS. The model explained 14% of the total variance.

**Conclusions:**

Indwelling urinary catheter placement status at admission, age, core transfer subscale score at admission, and difference in the CARE-C continence subscale score were identified as predictors of the LOS. The explanatory power of these predictors might be limited due to the regulations of Taiwan’s National Health Insurance.

## Introduction

Post-acute care (PAC), which refers to medical care services that support the individual’s recovery from acute illness or management of chronic disability, aims to enhance the functional status of patients discharged from acute medical wards [1]. Stroke is among the top five critical illnesses covered by the National Health Insurance (NHI) program, and patients with stroke account for the largest proportion of the population with disabilities needing continued medical, nursing, and rehabilitation services, which represent considerable medical costs [2, 3]. Since the launch of Taiwan’s NHI program in 1995, it has been acclaimed for its efficiency and effectiveness [4]. However, to relieve the heavy financial burden caused by the prolonged hospital stay of acute care patients with stroke, the NHI administration started to implement the PAC program for stroke in 2013, with the goal of developing a seamless system transfers of stroke patients from acute care units to subacute rehabilitation or long-term care facilities [3]. The Continuity Assessment Record and Evaluation (CARE) is a standard set of items developed by the US Centers for Medicare and Medicaid Services to evaluate the functional status of patients across various acute and PAC settings and determine resource utilization and medical expenses [2, 5]. The advantages of this tool include its nonproprietary feature, sensitivity to functional changes, multidimensionality, and reliability [6]. Given the lack of a generic and easy-to-use tool to evaluate the functional status of PAC patients in Taiwan, a Chinese version of the CARE item set (CARE-C) had been recently developed in an attempt to address this issue [2]. The reliability and validity of CARE-C in the assessment of functional outcomes among patients with stroke in Taiwan had been established in a previous study [2].

The length of hospital stay (LOS) is the principal predictive factor of medical expenses among variables that affect the total costs during hospitalization [7, 8]. Accurate estimation of the LOS is important for patients and their family and healthcare providers because it facilitates rehabilitation planning, resource distribution, and healthcare system administration [7, 8]. Various studies that aimed to identify the predictive factors of the LOS among stroke patients have been conducted. Considering that functional status recovery is the primary goal of PAC, the prediction of the LOS in this stage using a functional evaluation tool is not surprising [8, 9]. CARE-C has subtler grading and more comprehensive subscales than the other functional evaluation tools commonly used by previous studies, such as the Barthel Index and Functional Independence Measure (FIM). Additionally, the CARE-C data could be uniformly collected at PAC admission, which facilitates data accessibility. Furthermore, CARE is proved to be similar to the Minimal Data Set and FIM in terms of prospective payment determination [10]. Therefore, we hypothesize that the CARE-C subscale scores at admission could predict the LOS of patients in the PAC ward. This study aims to investigate the predictive power of CARE-C on the LOS of stroke patients in Taiwan.

## Materials and methods

### Patient population

**Fig 1:**
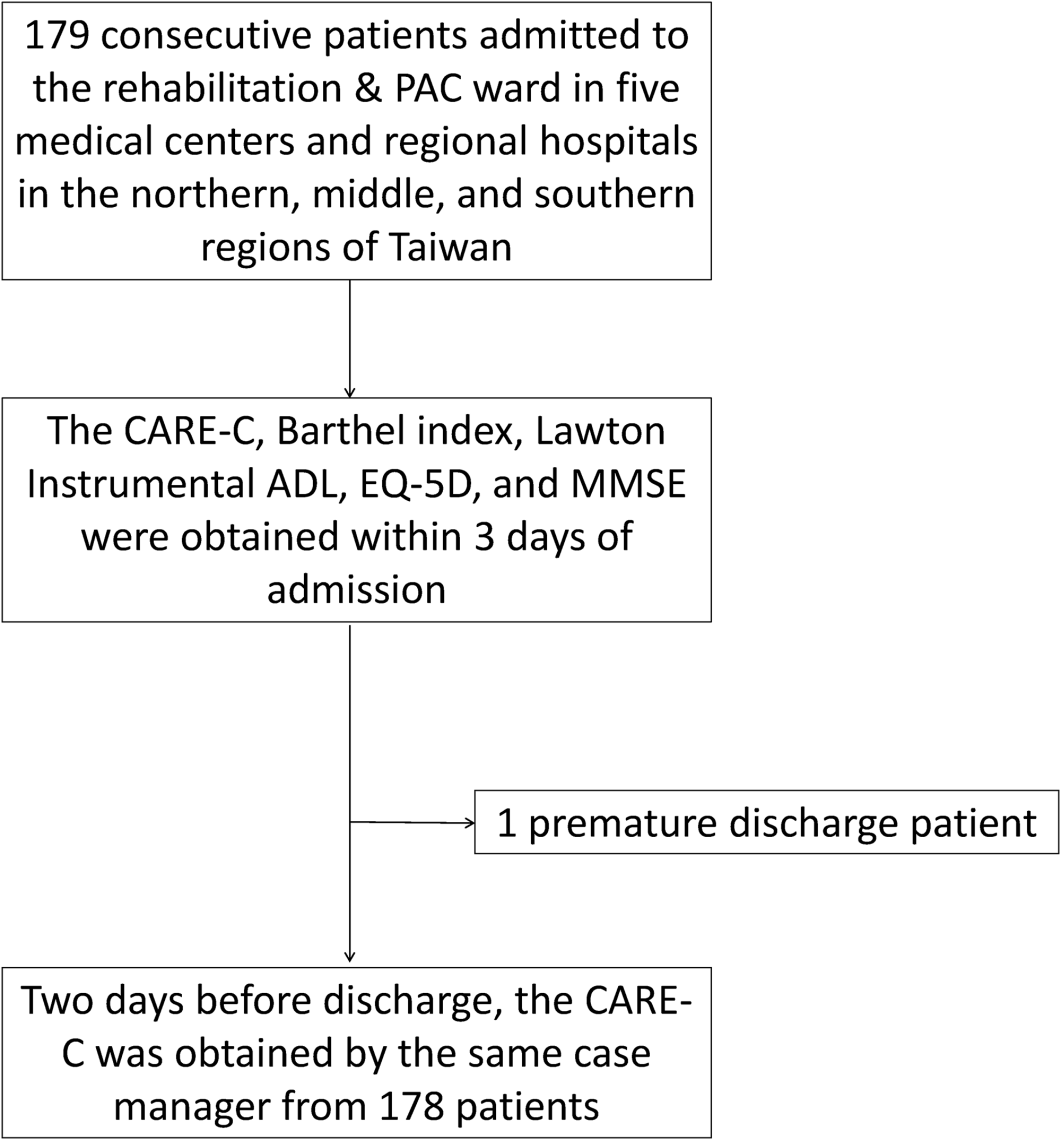
Flow chart of the patients and data recruitment. Abbreviation: ADL, Activities of Daily Living; EQ-5D, EuroQOL five-dimensions questionnaire; MMSE, Mini-Mental State Examination.

This prospective study involved patients who were admitted to the rehabilitation wards of five medical centers and regional hospitals in the northern, middle, and southern regions of Taiwan. During a 12-month period, consecutive patients were included in this study if they met the following criteria: (1) aged at least 20 years and (2) recently admitted (within 3 months) to the rehabilitation wards with a diagnosis of stroke or other central nervous system injuries, including traumatic brain and spinal cord injuries. Patients were excluded if they did not receive rehabilitation training during hospitalization. Fig 1 shows the recruitment flow chart. The study protocol was approved by the Institutional Review Board of the National Taiwan University Hospital (201406020RINB), and all participants provided their written informed consents prior to inclusion in this study.

### Data collection

After the patients were admitted to the target hospital, a group of case managers, who were nurses by profession, received a 1-day course on CARE-C assessment instructions and collected the data on CARE-C, Barthel Index, Lawton Instrumental Activities of Daily Living scale, EuroQOL Five-Dimension Questionnaire (EQ-5D), and Mini-Mental State Examination within 3 days of admission. These questionnaires record the actual, not the potential, performance of the patient’s functional status. If the patient cannot respond to the question well, his/her family or caregiver can answer the question for the patient.

The demographic data on age, sex, disease onset date, diagnosis, and nasogastric tube and indwelling urinary catheter placement statuses at admission were collected as well. Two days before discharge, the same case manager performed the second CARE-C assessment. CARE-C is composed of 52 items that were categorized into 11 subscales, which include cognition, delirium, disordered behavior, depression, pain, continence, perception, impairment, basic activities of daily living, transfer, and instrumental activities of daily living. Higher scores represent higher quality in the CARE-C rating. For example, participants with a higher depression subscale score are more depressed and those with a higher cognition subscale score have better cognitive function than those with lower scores in the respective subscales. The LOS was computed by subtracting the patient’s date of admission to the ward from the date of discharge.

### Statistical analysis

The minimum number of participants was computed by using an expected power and 2-sided type I error probability to detect a true association in the correlation coefficients between the null and alternative hypothesis levels. We assumed that the correlation value between the LOS and CARE-C subscale scores was 0.5 and 115 participants were needed for a predesignated alpha value of 0.05 and a power of 0.8 [11].

The descriptive statistics (mean and standard deviation) were calculated using Microsoft Office Excel 2003 (Microsoft, Seattle, WA, USA). Additionally, Pearson’s correlation analyses between the LOS and other variables were performed. The variables that showed a significant correlation with the LOS (p < 0.10) were included in subsequent multivariable stepwise linear regression modeling to identify the predictors of the LOS. In this model, the LOS was regarded as a dependent variable, and age, sex, diagnosis, tube placement status, CARE-C component score at admission, and score differences between admission and discharge (discharge score minus admission score, i.e., subscale progress) were regarded as independent variables. All statistical analyses were performed using SAS software (Version 9.2; SAS Institute, Cary, NC, USA) with a 5% significance level (p < 0.05).

## Results

This study enrolled 178 patients (66 women and 112 men, of whom 137, 23, and 18 had stroke, spinal cord injury, and traumatic brain injury, respectively), with a mean age of 61.92 years. Table 1 summarizes the demographic data of the participants. Table 2 shows the results of the Pearson’s correlation analysis for the LOS. Age, basic ADL and core transfer subscale scores, and Barthel Index score were negatively correlated with the LOS. Meanwhile, the disease onset, indwelling urinary catheter and nasogastric tube placement statuses, and EQ-5D score were positively correlated with the LOS. Generally, patients with earlier disease onset, more frequent catheterizations, poorer functional status, and younger age had longer LOS than their respective counterparts. Table 3 summarizes the results of multivariable stepwise linear regression analysis. Four variables were found to be the main explanatory factors for the LOS of PAC patients, which accounted for 14% of the total variance. Indwelling urinary catheter placement status at admission was a positive predictor of the LOS, whereas age, core transfer subscale score at admission, and difference in continence subscale score were negative predictors of the LOS.

**Table 1:** Demographic data of the patients recruited.

Variables	Participants
Number of subjects	178
Sex (M/F)	112/66
Age (years)	61.92 ± 15.63
Interval between onset and 1^st^ assessment (days)	41.29 ± 35.96
Bathel Index at admission	31.91 ± 23.61
MMSE at admission	17.26 ± 10.80
EQ-5D at admission	9.78 ± 1.99
Length of stay (days)	24.31 ± 6.51
Indwelling urinary catheter placement at admission (%)	29.8%
Nasogastric tube placement at admission (%)	27.0%

Values are mean ± SD.

Abbreviation: MMSE, Mini-Mental State Examination. EQ-5D, EuroQOL five dimensions questionnaire.

**Table 2:** Pearson correlation analysis for length of hospital stay.

Variable	Pearson Coefficient	P-value
Age	-0.156	0.038
Sex	0.053	0.480
Onset duration	0.284	<0.001
CARE subscale		
Cognition	-0.091	0.228
Delirium	-0.048	0.529
Disordered behavior	-0.036	0.632
Depression	-0.055	0.467
Pain	0.017	0.822
Continence	-0.128	0.089
Indwelling urinary catheter placement	0.211	0.005
Perception	-0.095	0.209
Nasogatric tube placement	0.173	0.021
Basic ADL	-0.192	0.010
Core transfer	-0.195	0.009
Instrumental ADL	-0.077	0.310
Continence progress	-0.127	0.091
Core transfer progress	-0.132	0.079
Criterion-Related scales		
Barthel index	-0.270	<0.001
L-IADL	-0.141	0.061
EQ-5D	0.176	0.018
MMSE	-0.115	0.127

Abbreviation:

ADL: Activities of Daily Living,

L-IADL: Lawton Instrumental Activities of Daily Living scale,

EQ-5D: EuroQOL five-dimensions questionnaire,

MMES: Mini-Mental State Examination

**Table 3:** Multivariable stepwise linear regression models for length of hospital stay (LOS).

	Coefficient (SE)	β	P-value
**Cohort (R**^**2**^ = **0.140)**			
Constant	30.476 (2.264)	-	<0.001
Age	-0.079 (0.030)	-0.189	0.010
Core transfer at admission	-0.192 (0.082)	-0.176	0.020
Indwelling urinary catheter placement at admission	3.427 (1.111)	0.241	0.002
Difference of continence subscale	-0.627 (0.233)	-0.203	0.008

Abbreviation: IADL, instrumental activities of daily living

## Discussion

Numerous studies that investigated the factors influencing the LOS of stroke patients are available in the literature. The determinants investigated in those studies include the patient’s functional measures, demographic data, number of comorbidities, neurologic deficits, socioeconomic status, and family support. A retrospective study conducted by Lin et al. showed that the modified Barthel Index and FIM were both significant tools in predicting the LOS, although they were limited by their reproducibility [8]. Additionally, Wee et al. performed a prospective study and found that balance, aphasia, number of impairments, and family support at admission had significant contributory predictive effects on the LOS [12]. Galski et al. reported that impaired higher-order cognitive functions, including comprehension, judgement, short-term verbal memory, and abstract thinking, extended the LOS [13]. A recent study by Yeh at al. showed that patients with older age, low socioeconomic status, and Charlson Comorbidity Index score of > 5 had reduced inpatient rehabilitation rates at 7–12 months after stroke [14]. The previously identified predictive factors significantly differ among studies, and this difference might be attributed to the study designs, different healthcare systems, and customs, culture, and heterogeneity of the study populations. This study is the first to explore the predictive power of CARE-C on the LOS of stroke patients.

In our study, age was identified as a negative predictor of the LOS, which is similar to the results of previous studies [14–16]. Elwood et al. hypothesized that rehabilitation teams may have lower functional recovery expectations in older patients than in younger ones, which resulted in shorter LOS in the former [15]. Furthermore, Atalay and Turhan suggested that older patients are more likely to be “prematurely discharged” due to increased transfers to acute hospitals, decreased ability to participate in rehabilitation, and family’s requests than their younger counterparts [17]. In Taiwan, the NHI regulations may have an effect on the relationship between age and LOS. For example, older patients have higher possibility of developing medical complications during the PAC program, such as urinary traction infection or pneumonia, than younger patients, which might lead to the suspension of rehabilitation program and transfer to acute medical ward for management. When the attending physician decides on the length of the admission period, older patients might also be deemed to have less potential to achieve rehabilitation progress than their younger counterparts, which leads to shorter LOS in this age group. Moreover, younger patients might have higher rehabilitation goals than older patients so that they can return to work. Thus, the former will have longer LOS than the latter to achieve this higher rehabilitation goals. Several studies had different findings with regard to the influence of age on the hospitalization duration. Some studies found that age had no effect on the LOS [17–20], whereas others showed that older age was a significant predictive factor affecting the LOS [21, 22]. However, the comorbidities of the patients included in these studies should be taken into account when interpreting the different influences of age on the LOS.

In our study, the core transfer subscale score at admission was a negative predictor of the LOS. This finding indicates that better core transfer performance at admission would lead to a shorter LOS. The core transfer items in CARE-C are as follows: lying to sitting on the bed side, sitting to standing, chair/bed-to-chair transfer, and toilet transfer. A previous study found that the mobility items of the FIM tool, including bed-to-chair, toilet transfer, and tub-to-shower transfers, had a significant negative correlation with the LOS of patients with stroke [23]. Patients with better performance in these activities were less dependent and more capable of leaving their beds and, therefore, had a higher chance of receiving rehabilitation via an outpatient clinic instead of an inpatient rehabilitation program than those with poorer performance.

Based on our results, urinary catheter placement at admission is a predictor of longer LOS, whereas improvement in continence status was a negative predictor of the LOS. Previous studies also reported that indwelling urinary catheter placement predicted lengthened hospital stay, increased risk of urinary tract infection, and poor outcome among patients at 3 months following acute stroke [24, 25]. Urinary retention or incontinence is a common complication in patients with acute stroke, and the placement of urinary catheter facilitates onward medical management by eliminating the need for frequent toileting or reducing the need for body cleaning and diaper changing by the nursing staff [24]. However, international guidelines suggested that the use of an indwelling urinary catheter should be avoided unless urinary retention or vulnerable skin condition is present [26]. In addition to the high risk of urinary tract infection, the use of a urinary catheter also hampers the participation of patients in the rehabilitation programs. The inconvenience of catheter changes outside the medical facility and development of possible complications were also sources of concern during discharge planning. Indwelling urinary catheters commonly remain in place after their placement without the knowledge of the attending physician [27]. Hence, physicians should keep in mind that urinary catheters should be removed once its use is no longer necessary. The CARE-C continence subscale comprised the bladder and bowel continence and swallowing ability. However, further analysis of the influence of swallowing ability on the LOS was not conducted in the present study.

In this study, the average LOS for patients with stroke in the rehabilitation ward was 24.31 ± 6.51 days. Despite the four significant predicting parameters, the variance explained by the linear regression model was considerably lower than that of other studies [12, 28]. This finding could partly be explained by the two regulations of the NHI program in Taiwan that are associated with the LOS. First, the patients have to pay 10% copayment when their LOS is < 30 days. However, this copayment rate will increase by 20% when the LOS is > 30 days. Second, the imbursement of patients with an LOS of > 30 days will be subjected to case-wise audits. The attending physician will take these two points into consideration when deciding the LOS of each patient. Grant et al. also found that the predictive factors had poor contribution on the LOS, with 20% of the total variance explained by their model. Additionally, they suggested that factors outside the functional, medical, and demographic patient characteristics, such as administrative and therapy-specific variables, have important influences on the LOS. Their inference is consistent with our finding. Considering that the multivariable linear regression analysis only accounted for a small portion of the total variance of the LOS, physicians and NHI administrators should be cautious when attempting to use a patient characteristic-based model to predict the LOS of patients in Taiwan.

The present study has several limitations that are worth noting. First, more patients should be recruited, and the associated comorbidities of the participants should be determined to evaluate the influence of other medical problems on the LOS. In our study, we recruited patients from the rehabilitation and PAC wards of the representative medical centers and regional hospitals from different regions of Taiwan. However, selection bias is inevitable with this non-all-inclusive recruitment process. Second, we only investigated the predictive power of CARE-C on the LOS among patients with stroke and central nervous system injuries in Taiwan. The predictive power of CARE-C on the LOS of other populations warrants future investigation. Third, CARE-C comprises 52 items, which are categorized into 11 subscales. The multivariable stepwise linear regression modeling was conducted by analyzing the score of each subscale, instead of every item. Therefore, caution should be exercised to prevent misinterpretation of the results (as we previously mentioned in the continence subscale in the Discussion section). Lastly, in Taiwan, medical decisions are considerably influenced by the NHI regulations, which leads to the small variance explained by the model. Future research that is designed without the restriction of these regulations is needed.

In summary, indwelling urinary catheter placement status at admission was a positive predictor of the LOS, whereas age, core transfer subscale score at admission, and improvements in continence subscale score were negative predictors of the LOS. The explanatory power of these predictors might be limited due to the regulations of Taiwan’s NHI. As clinicians in the PAC/rehabilitation ward, we should focus our treatment on transfer ability and bladder training to shorten the LOS. However, future longitudinal study is needed to prove the causal relationship between these variables.

## Supporting information

S1 Table STROBE statement. STROBE checklist of items.
(DOCX)
